# The Effects of Streptozotocin-Induced Diabetes and Insulin Treatment on Carnitine Biosynthesis and Renal Excretion

**DOI:** 10.3390/molecules26226872

**Published:** 2021-11-15

**Authors:** Aman Upadhyay, Kate E. Boyle, Tom L. Broderick

**Affiliations:** 1 Department of Biomedical Sciences, College of Graduate Studies, Midwestern University, Glendale, AZ 85308, USA; aupadhyay19@midwestern.edu; 2 Arizona College of Osteopathic Medicine, Midwestern University, Glendale, AZ 85308, USA; kate.boyle@midwestern.edu; 3 Laboratory of Diabetes and Exercise Metabolism, Department of Physiology, College of Graduate Studies, Midwestern University, Glendale, AZ 85308, USA

**Keywords:** diabetes, carnitine, biosynthesis, uptake, kidney, urine, insulin

## Abstract

Carnitine insufficiency is reported in type 1 diabetes mellitus. To determine whether this is accompanied by defects in biosynthesis and/or renal uptake, liver and kidney were obtained from male Sprague-Dawley rats with streptozotocin-induced diabetes. Diabetic rats exhibited the metabolic consequences of type 1 diabetes, including hypoinsulinemia, hyperglycemia, and increased urine output. Systemic hypocarnitinemia, expressed as free carnitine levels, was evident in the plasma, liver, and kidney of diabetic rats. Compared to control rats, the low free carnitine in the plasma of diabetic rats was accompanied by decreased expression of γ-butyrobetaine hydroxylase in liver and kidney, suggesting impaired carnitine biosynthesis. Expression of organic cation transporter-2 in kidney was also reduced, indicating impaired renal reabsorption, and confirmed by the presence of elevated levels of free carnitine in the urine of diabetic rats. Insulin treatment of diabetic rats reversed the plasma hypocarnitinemia, increased the free carnitine content in both kidney and liver, and prevented urinary losses of free carnitine. This was associated with increased expression of γ-butyrobetaine hydroxylase and organic cation transporter-2. The results of our study indicate that type 1 diabetes induced with streptozotocin disrupts carnitine biosynthesis and renal uptake mechanisms, leading to carnitine insufficiency. These aberrations in carnitine homeostasis are prevented with daily insulin treatment.

## 1. Introduction

Carnitine is essential in the transfer of long-chain fatty acids (FAs) across mitochondrial membranes where they undergo β-oxidation for energy production [1]. In addition to this critical role, carnitine acts as buffer of the CoA pool, excretes potentially toxic acyl-CoA residues from cells, and regulates the intramitochondrial ratio of acetyl CoA to CoA [2]. The importance of carnitine is supported by the observation that systemic carnitine deficiency (SCD) syndromes are associated with impaired mitochondrial β-oxidation of FAs, decreased energy production, lipid storage myopathy, decreased exercise capacity, and cardiomyopathy [3,4,5].

Carnitine is supplied from both dietary sources and endogenous synthesis. Endogenous biosynthesis of carnitine begins with the lysosomal degradation of lysine and methionine, producing trimethyllysine, followed by conversion to γ-butyrobetaine (BB) [6,7,8,9]. The final reaction involves the hydroxylation of BB to carnitine by γ-butyrobetaine hydroxylase (γ-BBH). In rats and humans, the liver and kidneys are the main sites for carnitine biosynthesis because all other tissues lack considerable γ-BBH activity [10]. Tissues lacking γ-BBH activity release BB into circulation for subsequent uptake by the liver and kidneys, where this precursor is converted to carnitine [11,12].

The Na+-dependent organic cation transporter (OCTN) facilitates both BB and carnitine uptake into tissues. The OCTN2 isoform is abundantly expressed in kidney and is critical in the reabsorption of carnitine from urine [13]. Loss of OCTN2 function causes SCD, indicating that expression of this carrier is critical in carnitine homeostasis [4,5]. Earlier studies examining the effects of type 1 diabetes mellitus (T1DM) on carnitine homeostasis in children revealed decreased serum levels of free carnitine (FC) and increased urine levels of FC, suggesting impaired renal handling of carnitine causing carnitine insufficiency [14,15,16,17,18,19]. Further carnitine losses via renal routes were observed in the presence of ketosis and ketoacidosis, indicating that loss of metabolic control disrupts carnitine homeostasis [20,21]. In support of this, insulin treatment minimized urinary carnitine losses, suggesting a beneficial role of this peptide in carnitine metabolism.

Hypocarnitinemia has been reported in models of T1DM, including the spontaneous diabetic-prone BB Wistar rat and the streptozotocin (STZ) diabetic rat. Common features of these models include decreased levels of FC in plasma, muscle, heart, and kidney as well as increased urinary FC loss [16,22,23,24,25]. A reduction in the levels of FC in the plasma and tissue of diabetic rats suggests that, in addition to defective renal mechanisms, carnitine biosynthesis is impaired. Low carnitine levels in STZ-induced diabetes have detrimental effects, including autonomic dysfunction, abnormal left ventricular remodeling, and cardiomyopathy [24,25,26]. In addition, carnitine deficiency in this model of diabetes is a factor linked to a reduction in glucose use, a metabolic aberration accompanied by increased ischemic injury to the heart and peripheral insulin resistance [27,28,29]. Chronic supplementation with carnitine has proven beneficial in several obese and non-obese diabetic models, resulting in increased glucose metabolism and insulin sensitivity, improved lipid profile, and increased cardiac ischemic tolerance [26,28,29,30,31]. Given that carnitine deficiency compromises metabolic function and is often reported in T1DM, a preliminary evaluation of the effects of diabetes on carnitine homeostasis was warranted. In this study, we examined the effects of STZ-induced diabetes and insulin treatment on γ-BBH and OCTN2 expression in liver and kidney as well as on FC levels in plasma, urine, and tissues in diabetic rats.

## 2. Results

### 2.1. The Effects of STZ-Induced Diabetes on Physical Characteristics

The effects of diabetes and insulin treatment on various physical and metabolic characteristics of rats are presented in Table 1. Compared to control rats, the final body weight was significantly lower in STZ-induced diabetic rats. In addition, total fat pad weight (expressed as the sum of inguinal, peritoneal, and visceral fat pads) was reduced in STZ diabetic rats compared to control rats. However, no differences between groups were observed in kidney and liver weight. Insulin treatment increased body weight and total fat pad weight compared to STZ diabetic rats.

The effects of diabetes and insulin treatment on plasma insulin, glucose, and non-esterified fatty acids (NEFAs) are also depicted in Table 1. As expected, injection of STZ in rats resulted in an insulinopenic state. This reduction in plasma insulin levels induced hyperglycemia and a significant increase in the levels of NEFAs in plasma. Compared to STZ rats, insulin treatment reduced plasma levels of glucose and NEFAs, resulting in an improvement of metabolic control.

Food and water consumption and urine output were also determined in STZ diabetic rats (Table 1). As expected, the insulinopenic state of STZ diabetic rats produced significant changes in these parameters. Food intake increased (approximately two-fold) in STZ rats compared to control rats and insulin treatment significantly reduced in food intake. Additionally reflecting the diabetic state, water intake increased (approximately six-fold) in STZ rats. However, unlike food intake, which was nearly normalized with insulin treatment, water intake remained elevated and was reduced only 2.3-fold with insulin treatment.

### 2.2. Plasma, Renal, and Hepatic Free Carnitine Levels in Diabetic Rats

**Figure 1:**
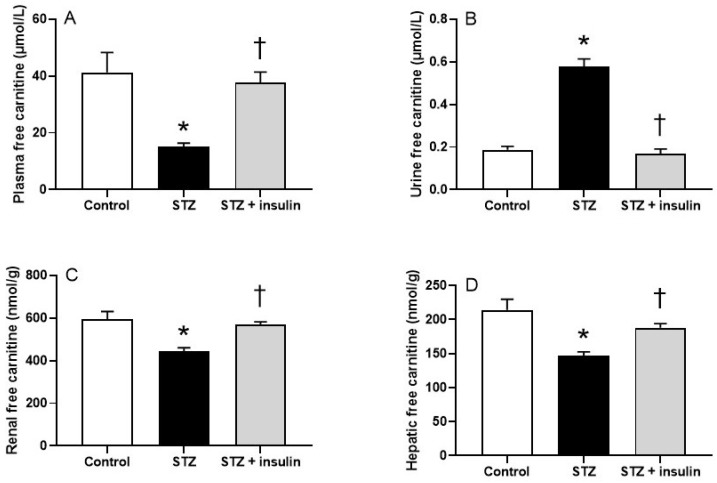
The effects of STZ diabetes and insulin treatment on FC levels in plasma (**A**), urine (**B**), kidney (**C**), and liver (**D**). FC, free carnitine; STZ, streptozotocin. Values are reported as mean ± SEM for 7–10 rats per group. * *p* < 0.05 compared to control rats. ^†^ *p* < 0.05 compared to STZ rats.

Figure 1 shows the alterations in carnitine homeostasis in STZ-induced diabetic rats. The carnitine content in plasma (Figure 1A), kidney (Figure 1C), and liver (Figure 1D), expressed as FC, was significantly decreased in STZ diabetic rats compared to control rats. To determine whether the low levels of plasma FC were associated with increased urinary excretion, FC levels were measured in urine. As shown in Figure 1B, levels of FC were significantly increased in the urine of STZ rats compared to control rats. Insulin treatment of diabetic rats restored the FC levels in plasma and decreased the FC levels in urine, suggesting that the low FC in plasma is explained by increased urinary loss. Levels of FC in kidney and liver were also improved with insulin treatment (Figure 1C,D).

### 2.3. Expression of Proteins Relating to Carnitine Biosynthesis and Uptake in Kidney of Diabetic Rats

**Figure 2:**
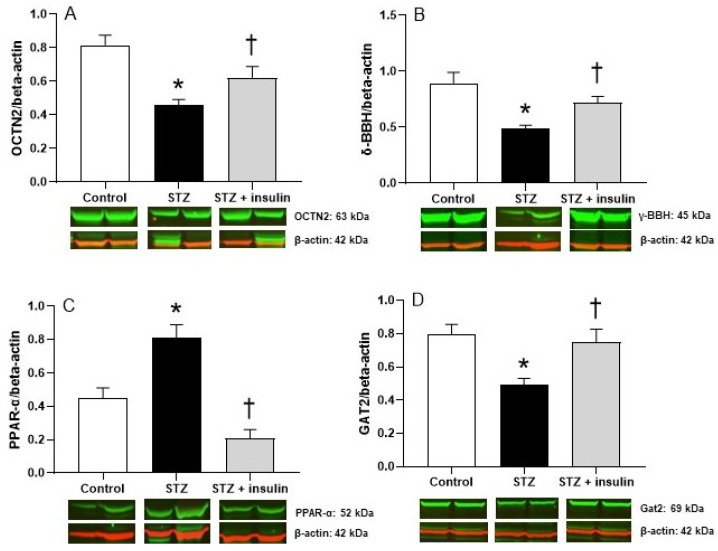
The effects of STZ-induced diabetes and insulin treatment on OCTN2 (**A**), γ-BBH (**B**), PPAR-α (**C**), and GAT2 (**D**) in rat kidney. STZ, streptozotocin; γ-BBH, gamma-butyrobetaine hydroxylase; OCTN2, novel organic anion transporter; PPAR-α, peroxisome proliferator-activated receptor-alpha; GAT2, gamma-aminobutyric acid transporter. Protein expression levels were normalized using β-actin as an internal control. Values are reported as mean ± SEM for 4–6 kidneys from two independent experiments. * *p* < 0.05 compared to control rats. ^†^ *p* < 0.05 compared to STZ rats.

The effects of the diabetic state on carnitine biosynthesis and uptake in kidney, expressed as protein levels of γ-BBH and OCTN2, respectively, are illustrated in Figure 2. Protein expression of γ-BBH decreased in the kidneys of STZ diabetic rats compared to control rats (Figure 2B). Protein content of OCTN2 was also reduced in the kidneys of rats (Figure 2A). These data suggest that the low levels of FC in the plasma of diabetic rats reflected the decreased expression of γ-BBH by the kidney. In addition, the elevated levels of FC in the urine of STZ diabetic rats (Figure 1C) can be explained by the lower protein content of OCTN2, which is essential in the reabsorption of FC from urine.

Since PPAR-α is known to regulate both γ-BBH and OCTN2 expression in tissues [32,33], protein expression of this transcription factor was measured in the kidneys of diabetic rats. Interestingly, as shown in Figure 2C, protein levels of PPAR-α increased in STZ-induced diabetic rats compared to control rats, indicating that PPAR-α expression is not correlated with either γ-BBH or OCTN2 expression in STZ-induced diabetes but is more closely associated with increased plasma NEFAs, its natural ligand (Table 1). Insulin treatment significantly increased γ-BBH and OCTN2 levels in STZ diabetic rats (Figure 2A,B), and these changes were consistent with the FC in plasma and in urine (Figure 1D). However, compared to untreated STZ diabetic rats, insulin treatment decreased protein levels of PPAR-α (Figure 2C). The effects of STZ-induced diabetes and insulin on sodium and chloride-dependent GABA transport 2 (Gat2), which mediates the transport of BB for carnitine synthesis [12,34], were also determined. As illustrated in Figure 2D, expression of Gat2 was significantly reduced in the kidneys of diabetic rats. With insulin treatment, expression of this transporter increased compared to the kidneys from uncontrolled diabetic rats, suggesting that insulin may be actively involved in the regulation of carnitine synthesis by stimulating BB uptake.

### 2.4. Expression of Proteins Relating to Carnitine Biosynthesis and Uptake in Liver of Diabetic Rats

**Figure 3:**
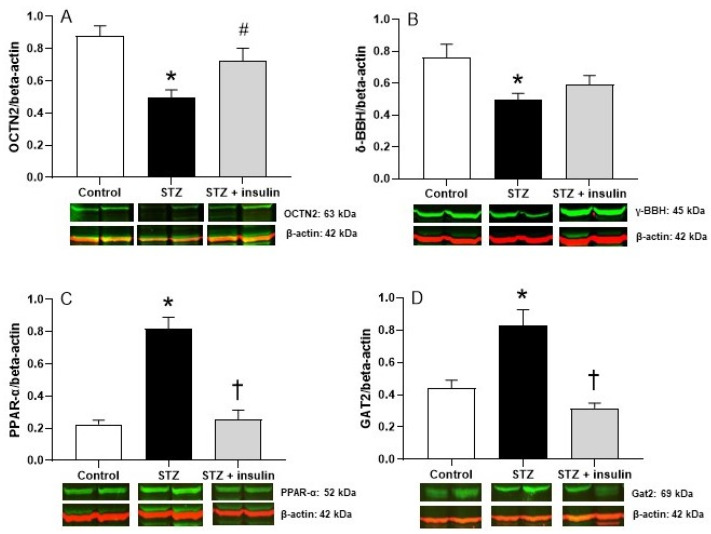
The effects of STZ-induced diabetes and insulin treatment on OCTN2 (**A**), γ-BBH (**B**), PPAR-α (**C**), and GAT2 (**D**) in rat liver. STZ, streptozotocin; γ-BBH, gamma-butyrobetaine hydroxylase; OCTN2, novel organic anion transporter; PPAR-α, peroxisome proliferator-activated receptor-alpha; GAT2, gamma-aminobutyric acid transporter. Protein expression levels were normalized using β-actin as an internal control. Values are reported as mean ± SEM for 4–6 kidneys from two independent experiments. * *p* < 0.05 compared to control rats. ^#^ *p* = 0.0507 compared to STZ rats (**A**). ^†^ *p* < 0.05 compared to STZ rats.

**Figure 4:**
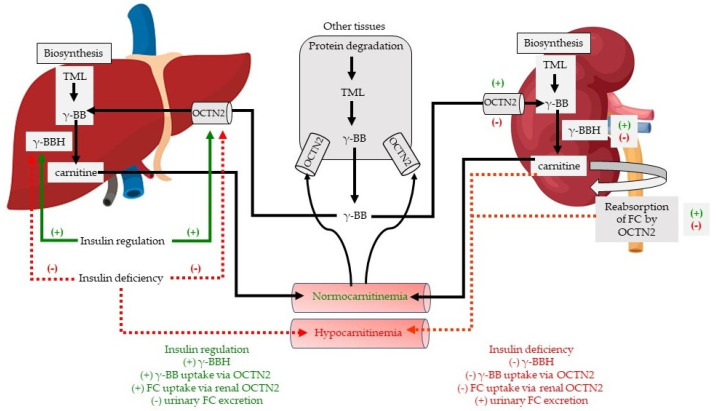
Proposed role of the effects of STZ-induced diabetes and insulin treatment on carnitine homeostasis. Under normal conditions, biosynthesis occurs primarily in the liver and kidney following protein degradation of lysine and methionine to TML [8]. TML is eventually converted to γ-BB. The final reaction in this pathway is the conversion of γ-BB to carnitine by γ-BBH. Carnitine is then released into circulation where it enters tissues against a concentration gradient via the OCTN2 transporter. In tissues that express low γ-BBH function, γ-BB is released into circulation and is actively taken up by the liver and kidney for conversion to carnitine. OCTN2 in kidney promotes reabsorption of FC from urine, thus preventing FC loss. Pancreatic islet β-cell necrosis by STZ results in an insulinopenic state and metabolic aberrations consistent with T1DM. In diabetic rats, we propose that insulinopenia decreases protein expression of γ-BBH and OCTN2 in liver and kidney, resulting in impaired biosynthesis and increased excretion of FC. Low γ-BBH reduces biosynthesis, while low OCTN2 enhances urinary losses of FC, both contributing to hypocarnitinemia. Daily insulin treatment, by stimulating synthesis of these proteins, prevents hypocarnitinemia. FC, free carnitine; γ-BB, gamma butyrobetaine; γ-BBH, gamma-butyrobetaine hydroxylase; OCTN, Na+-dependent organic cation transporter; TML, trimethyllysine; (+), stimulation; (−) decreased stimulation.

Expression of γ-BBH and OCTN2 in liver is shown in Figure 3. OCTN2 also increases BB uptake, the final precursor in the carnitine biosynthetic pathway for conversion to carnitine by γ-BBH. The effects of diabetes on γ-BBH and OCTN2 expression in liver closely mirror the changes observed in kidney. Protein levels of OCTN2 (Figure 3A) and γ-BBH (Figure 3B) decreased in the liver of STZ diabetic rats compared to control rats. Similarly, expression of the transcription factor PPAR-α increased in diabetic rats compared to control rats (Figure 3C). Insulin treatment of diabetic rats improved levels of OCTN2 and γ-BBH compared to STZ rats, and the effect of insulin was almost significant (*p* = 0.0507). However, the expression of PPAR-α was significantly reduced by insulin treatment of STZ rats (Figure 3C). Protein levels of Gat2 were increased in the liver of STZ diabetic rats compared to control rats. However, levels of Gat2 decreased with insulin treatment of STZ rats compared to untreated rats (Figure 3D). The effects of the diabetic state and insulin treatment on carnitine homeostasis is illustrated in Figure 4.

## 3. Discussion

In this study, we tested the hypothesis that the carnitine insufficiency observed in STZ diabetic rats resulted from impaired expression of hepatic and renal proteins relating to carnitine biosynthesis and uptake. After a period of eight weeks following the induction of diabetes, rats exhibited systemic hypocarnitinemia and the level of FC in the urine was elevated compared to control rats. These disturbances were associated with decreased protein expression of γ-BBH in the liver and kidney, the main organs in carnitine biosynthesis. Expression of OCTN2 in kidney, which has a high affinity for FC reabsorption, was also reduced in the diabetic rats, reflecting impaired reabsorption. Disruption of the carnitine status has also been reported in other models of diabetes, including diet-induced obese mouse and obese Zucker rat models of T2DM. The hypocarnitinemia in these models was also explained by decreased protein expression of γ-BBH in liver as well as decreased mRNA and protein expression of OCTN2 in skeletal muscle [35,36]. Taken together, it appears that carnitine insufficiency in experimental models of T1DM or T2DM is caused by defects in biosynthesis and uptake mechanisms. Due to the dramatic differences that exist in the phenotypes exhibited by these models of diabetes, it remains to be determined whether the hypocarnitinemia is due to chronic hyperglycemia, defective insulin signaling, or systemic inflammation.

In addition to the reduced protein expression of OCTN2 and γ-BBH detected in the tissues of STZ diabetic rats, we also demonstrated that expression of Gat2 was altered by the diabetic state. The Gat2 transport system, which is different from OCTN, is expressed in liver and kidney and transports BB for the final enzymatic conversion to carnitine [12,34,37]. Previous studies revealed that Gat2 contributes to more than half of BB uptake in isolated rat hepatocytes [34], suggesting that Gat2 could be of physiological relevance for carnitine biosynthesis. Western blot analysis showed decreased Gat2 expression in the kidney of diabetic rats, whereas expression of this transporter was increased in liver. The reasons why these reciprocal changes in Gat2 were observed are not clear and warrant further investigation. However, since the liver is the main organ in carnitine biosynthesis, it is reasonable to assume that the uptake of BB by Gat2 was enhanced to compensate for a reduction in biosynthesis. The observation that insulin treatment of diabetic rats restored Gat2 levels to control values and increased γ-BBH and FC contents in liver is consistent with this assumption.

The disturbances in the biosynthesis and renal uptake mechanisms of FC were prevented with daily insulin treatment. Insulin treatment was associated with increased expression of γ-BBH and OCTN2 and thus prevented the carnitine insufficiency and urinary losses in the STZ diabetic rats. Reversal of carnitine insufficiency with insulin treatment has been reported in children with T1DM. Compared to well-controlled insulin-treated diabetic patients, patients admitted for ketosis had decreased plasma FC, and further losses in FC were observed in newly diagnosed diabetics with frank acidosis and ketoacidosis [20,21]. Urinary excretion of FC in these patients was increased and reflected the degree of ketoacidosis. These disturbances were promptly reversed with insulin treatment, highlighting a critical role of insulin in plasma carnitine homeostasis and renal handling of FC in T1DM. Insulin treatment was also associated with reduced levels of short-chain acylcarnitines in urine. A reduction in short-chain derivatives comes largely at the expense of an increase in FC [21]. Since these alterations were observed in patients with a long history of T1DM, it is reasonable to assume that the acute disruptions in carnitine homeostasis reflected an acute hypoinsulemic state rather than a long-term complication of diabetes. Further supporting the role of insulin is the observation that mRNA expression of OCTN2 in skeletal muscle increased in the presence of insulin [38]. Although urinary loss of FC was prevented with insulin, further studies using isolated cell systems are warranted to determine whether insulin directly regulates the expression of OCTN2, thus preventing carnitine deficiency from renal routes. As a limitation of this study, the precise effects of insulin underlying its beneficial utility on carnitine biosynthesis in this model of T1DM were not determined. In addition to its role in glucose uptake and oxidation, insulin may enable activation of carnitine biosynthesis. This latter role is of functional importance because L-carnitine supplementation is known to stimulate glucose oxidation and improve functional recovery of diabetic hearts following ischemia [26,27,39].

Endogenous carnitine biosynthesis and renal uptake mechanisms are stimulated under conditions where NEFA levels are increased, such as exercise, fasting, and caloric restriction [40,41,42]. Release of NEFAs from adipose tissue activates PPAR-α, which in turn stimulates protein expression of γ-BBH and OCTN2 in hepatic and renal tissue [33]. This facilitates mitochondrial oxidation of fatty acids and prevents urinary losses. Evidence shows that expression of γ-BBH and OCTN2 is dependent on activation of PPAR-α since PPAR-α-null mice exhibit low OCTN2 expression and develop carnitine deficiency [32,43]. Based on these observations, we expected that this relationship would exist in the STZ diabetic rats. Although elevated NEFA level was associated with stimulation of PPAR-α expression in liver and kidney, this was not met by corresponding increases in protein levels of γ-BBH and OCTN2. However, our results are consistent with earlier studies which demonstrated that changes in OCTN2 expression do not correlate with PPAR-α levels. Low OCTN2 mRNA expression in the gastrocnemius muscle of obese mice was reported after exercise training [36]. In high fat-, high sugar-fed mice, OCTN2 expression in kidney increased after exercise training in the absence of detectable changes in PPAR-α expression [44].

We measured FC since it is the major fraction accounting for the total circulating carnitine pool in the plasma and its concentration is modified when rates of FA oxidation in tissues are elevated [45,46]. The esterified fraction of carnitine and the total content were not measured in the plasma and tissues of diabetic rats. Therefore, based on this limitation, it is not possible to determine whether total carnitine was decreased by the diabetic state or if a change in the ratio of FC to acylcarnitines occurred. However, evidence from early studies has indicated that a redistribution in the ratio of FC to acylcarnitines, favoring a reduction in FC, occurs in uncontrolled diabetes [15,19,20,21,47]. A similar redistribution of carnitine fractions was reported in short-term fasting, reflecting a state of elevated FA oxidation in tissues [45,46]. Based on these observations, it is possible that the decrease in plasma FC observed in the STZ diabetic rats was due to increased uptake by tissues to facilitate the disposal of NEFAs. The increase in FC and long-chain acylcarnitine in soleus muscle obtained from 10-day-old severely STZ-induced diabetic rats is consistent with this mechanism [47]. Increases in the carnitine content in plasma, liver, and soleus were also observed in 7-day-old STZ rats [16]. Interestingly, in both of these studies, the total carnitine content was increased above control levels in STZ diabetic rats, and insulin treatment restored total carnitine well into normal levels. Clearly, our results showing that insulin treatment increased the carnitine content in the plasma and tissues of STZ diabetic rats are not consistent with these earlier reports. The reasons for these apparent discrepancies are not known but may be explained, in part, by the severity and duration of the diabetes. The dose of STZ used to induce diabetes in our study was lower (55 mg/kg vs. 75–100 mg/kg) and the duration of diabetes was longer (8 weeks vs. 1–1.5 weeks).

## 4. Materials and Methods

### 4.1. Rat Model of Diabetes

The Midwestern University Research and Animal Care Committee approved this study. All animals used in this study were cared for in accordance with the recommendations in The Guide for the Care and Use of Laboratory Animals (National Institute of Health, Publ. 85–23, 1986). Male Sprague-Dawley (Charles River Laboratories, Wilmington, MA, USA) aged 2 months were divided into 3 groups (*n* = 7–10/group): control, diabetic, and diabetic with insulin treatment. Diabetes was induced in isoflurane-anesthetized rats with a single intraperitoneal injection of STZ (55 mg/kg, Sigma-Aldrich Chemicals, St-Louis, MO, USA) dissolved in 50 mM citrate buffer, pH 4.5. Control rats received an intraperitoneal injection of citrate buffer only. The diabetogenic effect of STZ consists of pancreatic β-cell necrosis, producing insulinopenia and resulting in hyperglycemia, polydipsia, increased urine output, and disrupted carnitine homeostasis. All these changes are consistent with the human condition of T1DM. Insulin (Insulin N, Novo Nordisk, Bagsvaerd, Denmark, 1–2 U/100 g) treatment was initiated after the confirmation of diabetes by measuring urine glucose content using glucose oxidase strips (Bayer, Diastix, Whippany, NJ, USA). Diabetic rats received insulin once per day in the morning for a period of 8 weeks. At the end of the study, diabetic rats received approximately 5–7 U/day based on body weight. Rats were provided food and water ad libitum and maintained in a room with an alternating twelve-hour light/dark cycle at a temperature of 22 °C.

### 4.2. Food and Water Intake and Urine Collection

Rats were placed in metabolic cages on week 6 of the study for a 24-h period to measure food and water intake, and then on week 7 for a period of 12 h to measure urine output. Volume was recorded and urine was aliquoted and stored at −80 °C for subsequent measurement of FC (Abcam, Cambridge, MA, USA).

### 4.3. Blood Sampling and Tissue Collection

After an overnight fast, rats were briefly gassed with CO_2_ and euthanized by cervical decapitation. Mixed blood was collected in chilled tubes containing EDTA and centrifuged at 12,000 rpm for 4 min. The plasma was removed and stored at −80 °C for measurement of glucose (Abcam, Cambridge, MA, USA), insulin (Alpco Diagnostics, Salem, NH, USA), non-esterified fatty acids (Abcam, Cambridge, MA, USA), and FC (Abcam, Cambridge, MA, USA) following the manufacturer’s guidelines and using a multi-mode microplate reader (Biotek Instruments Inc., model Synergy 2, Winooski, VT, USA). Livers and kidneys were excised, weighed, and snap frozen in liquid nitrogen. Inguinal, peritoneal, and visceral fat pads were carefully excised and weighed to determine total fat pad weight.

### 4.4. Western Blot Analysis

Liquid homogenization of the liver and kidney samples was performed, and samples were stored at −80 °C. Briefly, samples were analyzed for protein content (Tissue PE LB, G-Biosciences, St-Louis, MO, USA) and separated on 4–12% Bis-Tris gels at 150 volts for 1.5 h. Dry transfer was performed using the iBlot2 Dry Blotting system (Thermo Fisher Scientific, Waltham, MA, USA). Blots were incubated with primary antibody for OCTN2 (1:1000; Abcam Cambridge, MA, USA), BBH (1:1000; Abcam, Cambridge, MA, USA), PPAR-α (1:1000; Abcam Cambridge, MA, USA), and GAT2 (1:1000; Abcam, Cambridge, MA, USA) overnight at 4 °C. To probe for actin, blots were incubated with anti-actin primary antibody (1:5000; EMD Millipore, Billerica, MA, USA) for 1 h at room temperature. After washing, blots were incubated with the secondary antibody anti-rabbit immunoglobulin G (IgG) (heavy and light chains) DyLight (1:100,000; Cell Signaling Technology, Danvers, MA, USA) and, simultaneously, with anti-mouse IgG (H + L) DyLight (1:100,000; Cell Signaling Technology) for 1 h at room temperature. Images of membranes were obtained, with the abundance of all proteins of interest normalized to actin, which was the internal control. Band density was analyzed using Odyssey-Clx and Image Studio (LI-COR, Lincoln, NE, USA).

### 4.5. Statistical Analysis

Data are presented as mean ± SEM. Group mean differences were determined using ANOVA, followed by the Student–Newman–Keuls comparison for post hoc analysis. GraphPad Prism software was used for the statistical analysis (San Diego, CA, USA). A *p* value < 0.05 was considered significant.

## 5. Conclusions

Our study indicated that plasma FC levels decreased while urinary values increased in STZ diabetic rats. We found that these changes were associated with reduced expression of γ-BBH and OCTN2/Gat2, suggesting defects in biosynthesis and reabsorption, respectively. Insulin treatment increased the levels of FC in plasma, liver, and kidney and minimized urinary losses. Our results are the first to indicate that insulin treatment has a significant impact on carnitine homeostasis and may be of clinical importance in the treatment of type 1 diabetes.

## Figures and Tables

**Table 1:** The effects of STZ-induced diabetes and insulin treatment on physical and metabolic characteristics.

Parameter	Control	STZ-Induced Diabetes	STZ + Insulin
Body weight (g) Fat weight (g) Kidney weight (g) Liver weight (g) Plasma insulin (ng/mL) Plasma glucose (nmol/L) Plasma NEFA (µmol/L) Food intake (g) Water intake (mL) Urine output (mL)	423 ± 12 12.36 ± 1.03 3.08 ± 0.09 12.51 ± 0.65 1.42 ± 0.18 5.45 ± 0.75 433 ± 11 28.6 ± 2.1 42.9 ± 2.5 12.5 ± 3.3	250 ± 14 * 2.61 ± 0.70 * 4.90 ± 1.07 12.60 ± 1.07 0.42 ± 0.03 * 9.37 ± 0.78 * 481 ± 16 * 52.8 ± 3.9 * 257.2 ± 20.8 * 71.9 ± 2.1 *	410 ± 17 ^†^ 10.82 ± 1.50 ^†^ 3.54 ± 1.49 15.76 ± 1.73 0.72 ± 0.05 *^,†^ 5.20 ± 0.25 ^†^ 445 ± 9 ^†^ 37.2 ± 5.3 ^†^ 111.7 ± 19.5 ^†^ 12.8 ± 2.2 ^†^

Values are expressed as mean ± SEM for 7–10 rats per group. STZ, streptozotocin; NEFA, non-esterified fatty acid. Plasma assayed for glucose, insulin, and NEFA was collected in overnight-fasted rats. Food and water intake was measured over a 24-h period in rats placed in metabolic cages. Urine output was measured over a period of 12 h. * *p* < 0.05 compared to control rats. ^†^ *p* < 0.05 compared to STZ diabetic rats.

## Data Availability

Not applicable.
